# Combining M-FISH and Quantum Dot technology for fast chromosomal assignment of transgenic insertions

**DOI:** 10.1186/1472-6750-11-121

**Published:** 2011-12-13

**Authors:** Mohammed Yusuf, David LV Bauer, Daniel M Lipinski, Robert E MacLaren, Richard Wade-Martins, Kalim U Mir, Emanuela V Volpi

**Affiliations:** 1 Wellcome Trust Centre for Human Genetics, University of Oxford, Roosevelt Drive, Oxford, OX3 7BN, UK; 2 Nuffield Laboratory of Ophthalmology and Oxford Eye Hospital Biomedical Research Centre, University of Oxford, John Radcliffe Hospital, Oxford, OX3 9DU, UK; 3 Department of Physiology, Anatomy and Genetics, Oxford Parkinson's Disease Centre, University of Oxford, South Parks Road, Oxford, OX1 3QX, UK

## Abstract

**Background:**

Physical mapping of transgenic insertions by Fluorescence in situ Hybridization (FISH) is a reliable and cost-effective technique. Chromosomal assignment is commonly achieved either by concurrent G-banding or by a multi-color FISH approach consisting of iteratively co-hybridizing the transgenic sequence of interest with one or more chromosome-specific probes at a time, until the location of the transgenic insertion is identified.

**Results:**

Here we report a technical development for fast chromosomal assignment of transgenic insertions at the single cell level in mouse and rat models. This comprises a simplified 'single denaturation mixed hybridization' procedure that combines multi-color karyotyping by Multiplex FISH (M-FISH), for simultaneous and unambiguous identification of all chromosomes at once, and the use of a Quantum Dot (QD) conjugate for the transgene detection.

**Conclusions:**

Although the exploitation of the unique optical properties of QD nanocrystals, such as photo-stability and brightness, to improve FISH performance generally has been previously investigated, to our knowledge this is the first report of a purpose-designed molecular cytogenetic protocol in which the combined use of QDs and standard organic fluorophores is specifically tailored to assist gene transfer technology.

## Background

The identification and characterization of transgenic insertions within the host genome is considered good practice for a number of different reasons. For instance, it can verify whether a transgenic animal is either homozygous or hemizygous for the transgene, an important aspect of breeding strategies. It is also useful for discerning targeted integration events from random ones, particularly when inappropriate gene expression and/or unexpected phenotypes are observed and insertional inactivation of a host gene is suspected. Transgenic insertion mapping has recently led to the discovery of a novel locus on proximal chromosome 18 associated with a congenital abnormality of the brain structure in mouse [1], and has also enabled the finding of an association between a dominantly inherited cone degeneration in a mouse model and a locus on chromosome 10 orthologous to a genomic region linked to a number of inherited retinal disorders in humans [2].

Physical mapping of transgenic insertions by Fluorescence in situ Hybridization (FISH) in rodents is a well established and cost-effective technique which can be applied either independently for chromosomal assignment and zygosity status assessment [3,4], or as a validation route in cases where high-resolution mapping has previously been obtained by molecular methods, like DNA sequencing [5].

FISH mapping of transgenic insertions (and/or endogenous genomic sequences) in mouse and rat usually entails hybridizing a DNA probe, homologous to the transgenic sequence, on metaphase chromosome spreads obtained from short term fibroblast cultures from tail or ear biopsies. The procedure normally involves tissue dissociation - done either mechanically or enzymatically - and culture of the resulting fibroblasts for one or more weeks prior to harvesting [6].

Chromosomal assignment is typically achieved by simultaneous G-banding or by means of multi-color FISH protocols, whereby the transgenic probe is co-hybridized with one or more differently labeled chromosome-specific probes. While interpretation of G-banding patterns is laborious and can be particularly challenging for the less experienced investigator, FISH-based methods, especially with the help of specifically designed software for digital image analysis, are more user-friendly. However, according to the specification of the fluorescence imaging system used, the "transgenic probe plus single chromosome-specific probe" approach may require many consecutive FISH hybridizations to establish eventually the chromosomal location of the transgenic insertion(s).

It has been previously shown that fast and accurate chromosomal assignment of transgene insertions in mouse can be achieved by sequentially combining FISH mapping with Spectral Karyotyping (SKY) [7]. SKY is a FISH-based technique that makes use of multiple, differently labeled DNA "paints" (chromosome specific DNA libraries) and Spectral Cube/Interferometer technology to analyze the spectral signature of each image pixel and simultaneously visualize and identify all the chromosomes in a karyotype at once. The combined use of FISH and SKY for transgenic detection has also the potential to identify transgene-induced chromosomal rearrangements, and accordingly is recommended as the most suitable approach for detecting unexpected genetic events associated with transgenic technology [8].

In the present report we describe a technical development whereby even faster chromosomal assignment of transgenic insertions at the single cell level in mouse and rat can be obtained by an efficient, combined hybridization procedure for Multiplex FISH (M-FISH) and the detection of transgene insertions, the latter of which are easily labeled with ultra-bright Quantum Dot (QD) conjugates.

M-FISH is a protocol for multicolor karyotyping, different in design but similar in purpose to SKY, wherein a combinatorial labeling algorithm enables simultaneous identification of all chromosomes at once as well as the accurate delineation of chromosomal aberrations [9], an often challenging task in species like mouse and rat in which inter-homologous differences in chromosome morphology and banding patterns are often non-conspicuous.

Quantum Dot nanocrystals (QDs) are inorganic fluorophores, made of semiconductor materials, which are orders of magnitude brighter than organic fluorophores. They are also more photo-stable, with hardly any noticeable photo-bleaching. Crucially for multiplex labeling experiments, QDs are available in multiple resolvable colors and have narrow emission bands with minimal spectral overlap. While QDs have a broad excitation spectrum, the excitation range is the same for each class of QD regardless of its emission wavelength, which makes it possible for them to be simultaneously excited, but distinguished from other fluorophores in a single exposure. Although the use of QD technology in FISH analysis is a relatively new field of investigation, a number of independent research groups have already explored their possible applications to chromosomal studies [10-14]. However, to our knowledge this is the first attempt at combining QD technology with multicolor karyotyping by M-FISH for physical mapping purposes. It should be noted that both M-FISH probes for mouse and rat, and QD-conjugated antibodies and affinity molecules such as Streptavidin are now commercially available.

## Results and discussion

**Figure 1:**
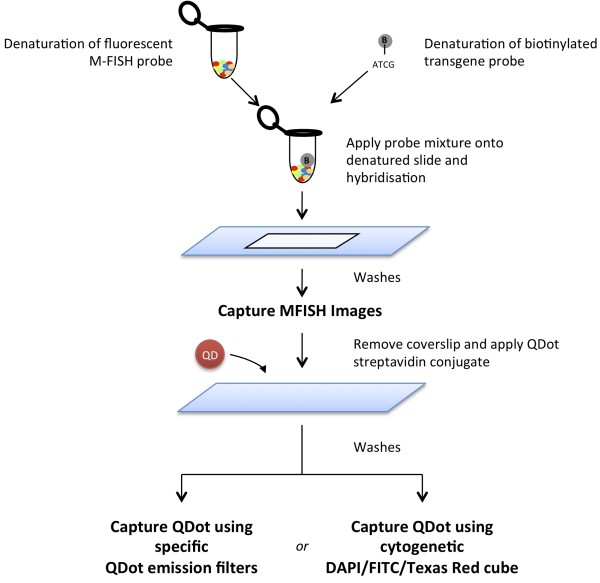
**Workflow for combined M-FISH and transgene detection using QDs**. The M-FISH probe set and the Biotinylated transgene probe are denatured and applied together in a "single step" hybridization.

We have devised an improved method for mapping of transgenic insertions in mouse and rat models. This procedure, which is described in detail in the Materials and Methods section, relies on M-FISH analysis for fast and accurate chromosomal identification in rodents, and differs from similar, previously described multicolor FISH-based mapping protocols for mixing in a simple, more efficient "single-step" hybridization the multiplex probe for the simultaneous recognition of all chromosomes and the transgene-specific probe for the detection of the integrated exogenous sequence (Figure 1). This development was accomplished by combining the use of organic fluorophores and QD nanocrystals in the same FISH experiment.

The chemical and spectral properties of QD are crucially different from those of other fluorophores, allowing for a critical degree of flexibility in the experimental and analytical steps. Briefly, the technique consists of co-hybridizing the directly labeled M-FISH multiplex probe for the animal species under investigation together and the indirectly labeled transgenic probe to the chromosome preparation, previously fixed on slide. Each of the chromosome-specific "paints" in the commercially obtainable multiplex or M-FISH probe is labeled with a finite number (five in our case) of distinct organic fluorophores (spectrally compatible to DEAC, FITC, Spectrum Orange™, Texas Red^® ^and Cy™5) in a unique combinatorial fashion, while the single transgenic probe is labeled in-house with a reporter molecule (Biotin in our case). Following the probe manufacturer's instructions, after 48 hours incubation at 37°C, post-hybridization washes and DAPI counterstaining, the slides are analyzed on the microscope. No detection step is required at this stage as the M-FISH probes are directly labeled. The initial goal is simply to acquire a number of informative M-FISH images (complete and well-spread metaphases) for either simultaneous or subsequent karyotype analysis. Following image capture, the slides are briefly washed and detection of the Biotin-tagged transgene carried out at once with QD conjugated-Streptavidin. Unlike in previously described protocols, there is no need for re-denaturation of the specimen and further overnight incubation (the transgene probe has been already hybridized), and there is also no need for "bleaching out" the M-FISH probes fluorescence (there is negligible interference in fluorescence emission between the M-FISH probes and the QD). Using the previously acquired M-FISH images as a reference, metaphases of interest are traced back and new images captured using the required filter for visualization of the specific QD emission.

The requirement for two separate imaging steps is dictated by the current specifications of the optical setup and specialized software for M-FISH analysis. M-FISH relies on a multiplex probe set of chromosome specific "paints", each labeled with a finite number of spectrally distinct fluorophores, and a set of filters for achieving optimal color separation for all fluorophores employed. If a combined imaging strategy is applied the QD fluorescence properties of broad excitation and strong emission end up interfering with the color separation and, as a result, scramble the automated decoding of the different labeling combinations, which is mandatory for the rapid M-FISH karyotyping. While we cannot exclude that these current hardware and software "limitations" might be addressed and resolved in the near future by the interested parties, two separate imaging steps - that is M-FISH imaging to be followed by QD detection - are presently the safest route to achieve clear-cut results with our combined M-FISH/QD protocol.

**Figure 2:**
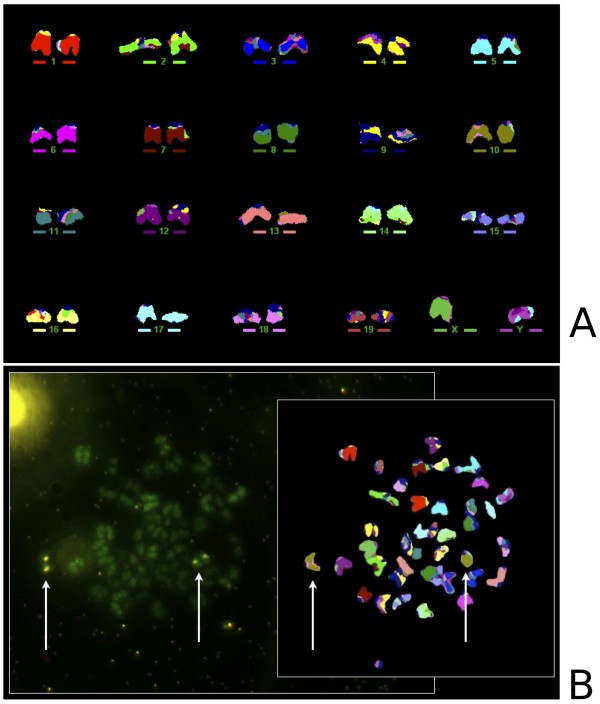
**Combined M-FISH analysis and QD detection allows chromosomal assignment of transgenic insertions in a mouse model**. A) Multicolor karyotype; B) Aligning the specific QD 655 and M-FISH metaphase spread images allows identification of chromosome 10 (pseudo-colored in brown by the M-FISH karyotyping software) as the chromosome holding the transgenic insertion and confirms this specific mouse model to be homozygous for the insertion (transgene presents on both homologs).

**Figure 3:**
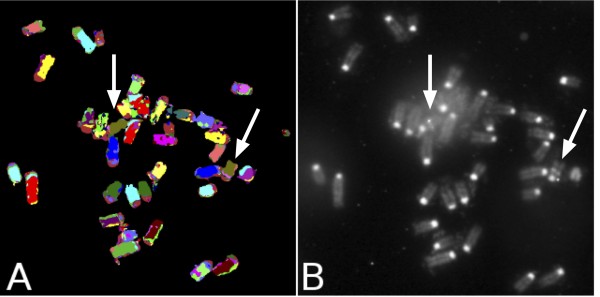
**Transgene signal as detected by QD 655 can be identified using a standard cytogenetic workstation**. A) M-FISH metaphase spread allows chromosome identification; B) QD 655 signal bleeds through the corresponding DAPI image taken on a cytogenetic workstation with a DAPI/FITC/Texas Red multiband beam-splitter and emission filter. Through the ocular the indicated signals appear red against the blue DAPI chromosomal stain.

We have successfully applied this procedure for the transgenic insertion mapping and zygosity status evaluation in the B6.Cg-Tg(OPN1LW-EGFP) mouse model [2]. To identify the chromosome holding the transgenic insertion(s) in the mouse model, a clone for the OPN1LW gene and its upstream region from a human fosmid library was labeled with Biotin and, after co-hybridization with the M-FISH probe, subsequently detected with a QD 655 Streptavidin conjugate (Invitrogen). As well as carrying out image acquisition in sequence on two different microscopes, specifically a CytoVision^® ^system equipped with a set of filters and interface software for M-FISH karyotyping (Leica Microsystems) and a Nikon TE-2000-E fluorescence microscope specifically equipped with QD filters from Chroma (Figure 2), most conveniently, we were able to observe the QD 655 emission on the CytoVision^® ^FISH workstation alone, using a DAPI/FITC/Texas Red triple filter set (multichroic and emission) under DAPI excitation (Figure 3). While the emission spectrum of QD 655 only overlaps partially with the emission window of Texas Red (part of the triple filter), the QD brightness is so high that it efficiently bleeds through into the emission channel and appears as ultra-bright points of light against the stained chromosomes on the monochrome CCD camera image. When observed through the ocular, the red QD 655 is easily distinguished from the blue DAPI background by eye and annotations can be made onto the monochrome images.

While the strength of the QD bleed-through may initially appear surprising, its origin lies in the brightness of the QD and their ability to efficiently absorb light at UV wavelengths: as a comparison, the molar absorbance of QD 655 is nearly 350 times that of DAPI at 350 nm and more than 135,700 times at 405 nm (data from Molecular Probes: The Handbook, 11^th ^Edition, http://www.invitrogen.com/site/us/en/home/References/Molecular-Probes-The-Handbook.html). When all the components of the microscopy system (lamp & filters) are taken into account with the QD and DAPI optical properties using a web-software tool developed by C Boswell and G McNamara at the University of Arizona http://www.mcb.arizona.edu/ipc/fret/index.html, the light output of QD 655 is calculated to be 271 times greater than DAPI when using a triple DAPI/FITC/Texas Red filter set under DAPI excitation (Table 1).

**Table 1:** Relative brightness of DAPI and QDs using various fluorescence filter set configurations

Filter Set Configuration	A	B	C	Modified C
**Filters**				
Excitation	DAPI	DAPI	DAPI	DAPI
Dichroic	Triple	Triple	DAPI	DAPI
Emission	Triple	DAPI	DAPI	QDot 655

**Light Output**				
DAPI	135	135	269	0
QDot 585	6,300	252	42	490
QDot 655	36,637	983	1,747	27,846

**Ratio QDot 655: DAPI**	**271**	**7.3**	**6.5**	

Light output values take the emission lines of the mercury arc lamp, filter sets, and dye absorbance and emission spectra into account to determine the light output of particular fluorophores given a particular optical configuration. Note that a "Triple" filter and dichroic refers to a standard multichroic or emission filter with multiple bandpasses for DAPI/FITC/TRITC, typical on a cytogenetics workstation. Light output values were obtained using "Fluorescent Spectra: An Interactive Exploratory Database", a web-software tool developed by C Boswell and G McNamara http://www.mcb.arizona.edu/ipc/fret/.

**Figure 4:**
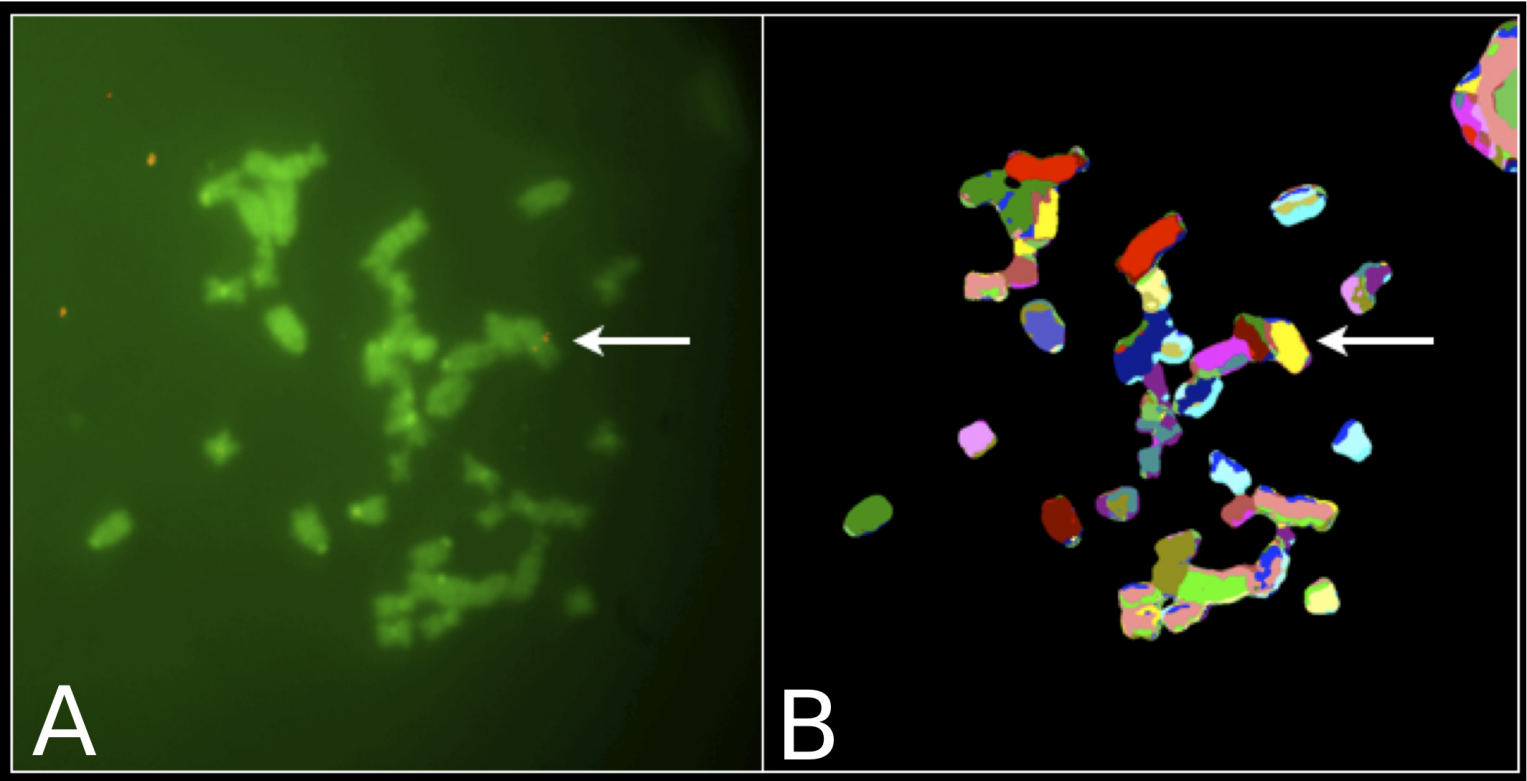
**Combined M-FISH analysis and QD detection allows chromosomal assignment of transgenic insertions in a rat model**. Aligning the QD 585 detection image (A) and M-FISH metaphase spread image (B) again allows identification of chromosome 6 (pseudo-colored in yellow by the M-FISH karyotyping software) as the chromosome holding the transgenic insertion and confirms this specific rat model to be hemizygous for the insertion (transgene presents only on one of the two homologs).

Visualization of the transgenic insertion and zygosity status evaluation in the second animal model under investigation - a transgenic rat carrying a human bacterial artificial chromosome (BAC) - was carried out by detecting the Biotin labeled BAC probe as described above, but using a different QD Streptavidin conjugate, namely the QD 585 (Invitrogen). Using this specific conjugate, it was not possible to unequivocally visualize the QD signal using the DAPI/FITC/Texas Red triple filter set under DAPI excitation. This was probably due both to the lower extinction coefficient of QD 585 as compared to QD 655 and to the spectral properties of the triple filter set used, which blocks light emitted at 585 nm to a large degree. Image acquisition could only be carried out in sequence on the two different systems, namely the fluorescence microscope equipped with filters and interface software for M-FISH analysis and the fluorescence microscope equipped with the specific QD filter (Figure 4). Therefore for the most rapid method of transgene localization, using filters sets commonly used by a typical cytogenetics laboratory, we would recommend using QD 655.

The same parameters used for standard FISH signal interpretation with organic fluorophores should be applied to the QD images. It is usually possible to discriminate true signals from false positives through experience and logical deduction. To start with, the signal/noise ratio should be reasonable. Indeed, if the fluorescent background is too high (either diffused, grainy fluorescence all over the specimens and/or bright speckles all over the slides) and no single signal stands out, the slides should go through additional post-hybridization or post-detection washes, and should effectively be reprocessed for detection. As in conventional FISH analysis, fluorescent signals not lying on top of chromosomes, and sitting outside the chromosome or nuclei contours, should be deemed aspecific background and excluded from the signal count. For confident chromosomal assignment the investigator should expect a doublet signal (two spots or "doublet" per chromosome, one for each chromatid) to be consistently found on the same chromosome in a minimum of 10-15 M-FISH metaphases per transgenic sample. For instance, in our samples, we observed 89% (25/28) of transgenic signals to be present as "doublets", while the remaining 11% (3/28) appeared as "singlets" (one spot per chromosome). Metaphases with "null" signals were not imaged.

**Figure 5:**
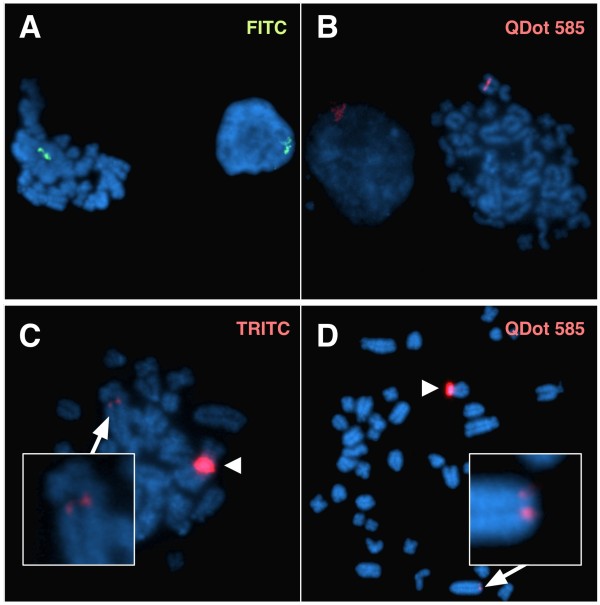
**Performance of QDs vs Organic Fluorophores**. Parallel hybridizations on the hemyzygous rat model using the same chromosomal preparation and the same biotin-labeled transgenic probe (A and B) show fluorescent signals comparable in size and brightness after indirect detection with either FITC (green) or QD585 (red) conjugated streptavidin. With both QD detection and organic fluorophore detection, it is also possible to estimate whether the insertion site is likely to contain one or multiple copies of the transgene (C and D). In this specific model, the relative size of two different insertions within the same karyotype suggests one of the two insertion sites (arrowhead) to contain multiple copies of the transgene. The inset shows a single copy transgene integration on a different chromosome.

The size and brightness of the transgenic signal as detected by QD conjugate after the M-FISH analysis both on metaphase chromosomes and interphase nuclei was very much similar to the signal for the same transgenic insertion detected by FITC- or TRITC-avidin in standard FISH mapping experiments that were carried out as controls (Figure 5). With both QD and organic fluorophore detection, it is also possible to estimate whether the insertion site is likely to contain one or multiple copies of the insert, either by taking in consideration the expected size of the insert or by direct visual comparison of the transgenic insertion size to the corresponding endogenous FISH signal on the control species from which the transgene originates, or by direct visual comparison of two different insertions within the same karyotype (Figure 5).

## Conclusions

We describe a simple "single denaturation mixed hybridization" procedure combining multi-color karyotyping by Multiplex FISH (M-FISH), for simultaneous and unambiguous identification of all chromosomes at once, and the use of a Quantum Dot (QD) conjugate for fast chromosomal assignment of transgenic insertions at the single cell level in mouse and rat models. To our knowledge this is the first report of a purpose-designed molecular cytogenetic protocol in which the combined use of QDs and standard organic fluorophores is specifically tailored to assist gene transfer technology.

## Methods

Chromosome slides for FISH analysis were obtained from short-term rodent fibroblast cultures established from ear explants as previously described [6]. Briefly, the cells were cultured in Dulbecco's Modified Eagle's Medium (DMEM) supplemented with 10% foetal bovine serum (FBS) and 1% L-Glutamine at 37°C in a 5% CO_2 _incubator. One hour before harvesting, the cells were treated with Colcemid (Gibco BRL) at a final concentration of 0.2 μg/ml. After trypsinization the cells were resuspended in pre-warmed hypotonic solution (0.075 M KCl or alternatively 0.034 M KCl, 0.017 M trisodium citrate) at 37°C for 5 minutes and fixed in three changes of 3:1 methanol: acetic acid. Slides were prepared by dropping a small volume of suspension onto glass microscope slides and allowed to air-dry overnight.

To identify the chromosome(s) holding the transgenic insertion in the mouse model, a clone for the OPN1LW gene and its promoter region from a human fosmid library was labeled by nick-translation (Abbott Molecular) with Biotin-16-dUTP (Roche) and co-hybridized with a panel of mouse M-FISH probes (21Xmouse mFISH probe kit by MetaSystems). To identify the chromosome(s) holding the transgenic BAC insertion in the rat lines the BAC clone was labeled as above and co-hybridized with a panel of rat M-FISH probes (22Xrat mFISH probe kit by MetaSystems).

The transgenic clones were ethanol precipitated in a mix of Salmon testis DNA (Gibco BRL), Escherichia coli tRNA (Boehringer) and 3 M sodium acetate. They were then dried on a heating block at 60°C with a 50X excess of Human Cot-1 DNA (Invitrogen) and resuspended at 20 ng/μl in hybridization solution (50% formamide, 10% dextran sulphate, 2XSSC). The probes were denatured at 72°C for 5 minutes and pre-annealed at 37°C for 30 minutes before mixing with the mFISH probe, which was denatured at 75°C for 5 minutes and pre-annealed at 37°C for 30 minutes. The combined transgenic probe and M-FISH probe mixture was then applied to the slides. The slides had previously been incubated in 2XSSC at 70°C for 30 minutes, left to cool down in the coplin jar at room temperature and then transferred in 0.1XSSC for one minute. They had then been denatured in 0.07 N NaOH at room temperature for 1 minute, quenched in 0.1XSSC at 4°C and 2XSSC at 4°C for 1 minute each, dehydrated in an ethanol series and left to air dry. Following hybridization, the slides were washed using standard M-FISH washes, more precisely 0.4XSSC at 72°C for 2 minutes, followed by a wash in 2XSSC, 0.05% Tween20 at 42°C for 30 seconds. The slides were mounted with DAPI (4',6-diamidino-2-phenylindole)/Antifade (250 ng DAPI counterstain/ml antifade) recommended for the Metasystems 21Xmouse and 22Xrat probe kits.

M-FISH image capture and karyotype analysis were carried out on a CytoVision^® ^system (Leica Microsystems) consisting of an Olympus BX-51 epifluorescence microscope coupled to a JAI CVM4+ CCD camera. Imaging of DAPI, FITC and Texas Red was performed using standard triple band multichroic and emission filters (83500 multi-band set, Chroma), with specific excitation filters for DAPI, FITC and Texas Red mounted in an excitation filter wheel. Specific sets for Spectrum Aqua, Spectrum Gold, and Far Red were also mounted and image acquisition, analysis and karyotyping were carried out with the CytoVision^® ^Genus v7.1 software. After M-FISH capturing, coverslips were gently removed by dipping the slides in a coplin jar with 2X SSC, followed by one or two washes at room temperature for 2 minutes. Particular care was taken to avoid accidental damage to the specimens during this delicate phase of the protocol. Biotin-labeled transgenic probes were then detected by incubation at 45°C for 30 minutes with Streptavidin-QD Conjugates (either 585 or 655 nm), diluted 1:100 in QD Incubation Buffer (2% BSA in 50 mM borate pH 8.3 with 0.05% Sodium Azide, Invitrogen). After 30 minutes the slides were washed once for 5 min in 1 × PBS, 0.5% Tween 20 at 42°C. The slides were mounted with DAPI Antifade (Vector Laboratories).

**Figure 6:**
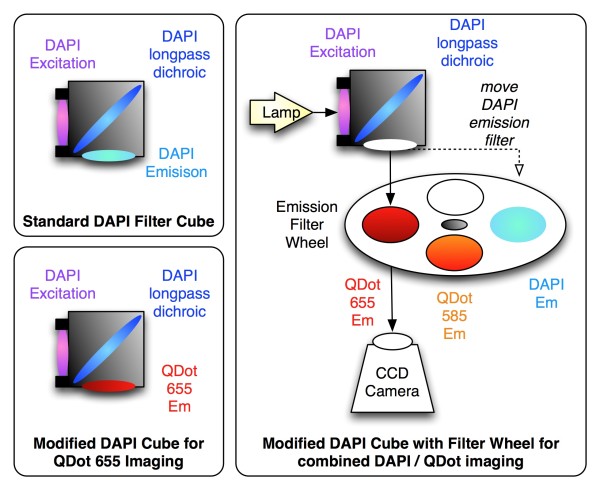
**A standard DAPI filter set can be easily modified to specifically image QDs**. Since both DAPI and QDs are efficiently excited by the DAPI excitation filter and well-split by a DAPI long-pass dichroic (such as 475dcxru), the DAPI emission filter can be replaced with a specific QD 655 emission filter (such as D655/40) to isolate the emission signal from the QD alone. For greater flexibility, multiple emission filters may be mounted in an external emission filter wheel.

Imaging of specific QD conjugates was performed on a Nikon TE-2000-E inverted fluorescence microscope with QD filters from Chroma (32000 Series) consisting of a wide-band UV excitation filter (E460SPUVv2), 475 nm dichroic mirror (475dcxru) and specific narrow-band emission filters for either QD 585 (D585/40) or QD 655 (D655/40). We were also able to use a standard DAPI cube (Nikon) to specifically image QD by leaving the excitation filter and dichroic in the cube, and replacing the DAPI emission filter with one of the specific QD filters (Figure 6). This process was simplified by placing the emission filters in an external filter wheel (Prior Scientific) to allow rapid switching between QD and DAPI imaging. Images were acquired with an EM-CCD camera (Hamamatsu Photonics, model C9100-13) controlled by MetaMorph software (Molecular Devices), which was also used to generate color overlay images.

Most conveniently for molecular cytogenetics laboratories that happen to be equipped only with a standard FISH analysis workstation, we were also able to visualize the QD 655 emission using the DAPI/FITC/Texas Red triple filter set under DAPI excitation on our CytoVison^® ^system as the QD brightness is so high that it efficiently bleeds through into the DAPI emission channel and appears as bright points of light against the stained chromosomes on the monochrome CCD camera image. When observed through the ocular, the red QD 655 is easily distinguished from the blue DAPI background by eye and annotations can be handily made onto the monochrome images.

## Authors' contributions

MY conceived the study, and carried out most of the experiments and the microscopy analysis. DLVB contributed to the design of the study, the microscopy analysis, the interpretation of the data and the manuscript drafting. DML carried out some of the experiments and revised the manuscript. REM-L and RW-M provided the transgenic models and revised the manuscript. KUM contributed to the design of the study, the data analysis and interpretation, and revised the manuscript. EVV contributed to the design of the study, the data analysis and interpretation, and drafted the manuscript. All Authors read and approved the final manuscript.
